# 2-[(4-Meth­oxy­benz­yl)imino­meth­yl]phenol

**DOI:** 10.1107/S1600536810048282

**Published:** 2010-11-24

**Authors:** Chuttree Phurat, Thapong Theerawattananond, Nongnuj Muangsin

**Affiliations:** a Research Centre of Bioorganic Chemistry, Department of Chemistry, Faculty of Science, Chulalongkorn University, Bangkok, 10330, Thailand

## Abstract

In the title Schiff base compound, C_15_H_15_NO_2_, prepared from 4-meth­oxy­benzyl­amine and salicyl­aldehyde, an intra­molecular O—H⋯N hydrogen bonds influences the mol­ecular conformation; the two aromatic rings form a dihedral angle of 73.5 (1)°. In the crystal, weak inter­molecular C—H⋯O inter­actions link the mol­ecules into chains propagating in [010].

## Related literature

For background to Schiff base ligands and their biological activity, see: Adsule *et al.* (2006 ▶); Karthikeyan *et al.* (2006 ▶). For related structures, see: Phurat *et al.* (2010 ▶); Tariq *et al.* (2010 ▶); Khalaji & Simpson (2009 ▶). For the graph-set analysis of hydrogen-bond patterns, see: Bernstein *et al.* (1995 ▶). For details of the synthesis, see: Phurat *et al.* (2010 ▶); Kannappan *et al.* (2005 ▶).
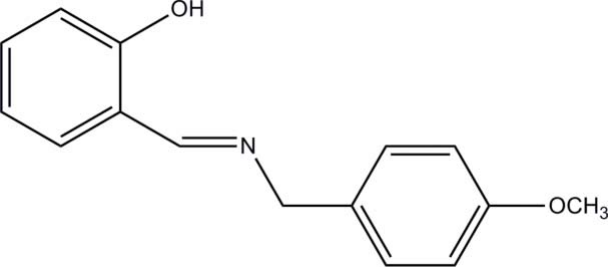


## Experimental

### 

#### Crystal data

C_15_H_15_NO_2_
*M* *_r_* = 241.28
Orthorhombic, 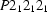

*a* = 5.7190 (8) Å
*b* = 12.7229 (19) Å
*c* = 17.936 (3) Å
*V* = 1305.0 (3) Å^3^
*Z* = 4
Mo *K*α radiation
μ = 0.08 mm^−1^
*T* = 293 K
0.3 × 0.18 × 0.04 mm

#### Data collection

Bruker SMART APEXII CCD area-detector diffractometer
5776 measured reflections
1573 independent reflections
1177 reflections with *I* > 2σ(*I*)
*R*_int_ = 0.040

#### Refinement

*R*[*F* ^2^ > 2σ(*F* ^2^)] = 0.051
*wR*(*F* ^2^) = 0.162
*S* = 1.13
1573 reflections
164 parameters
H-atom parameters constrained
Δρ_max_ = 0.15 e Å^−3^
Δρ_min_ = −0.16 e Å^−3^

### 

Data collection: *APEX2* (Bruker, 2008 ▶); cell refinement: *SAINT* (Bruker, 2008 ▶); data reduction: *SAINT*; program(s) used to solve structure: *SHELXS97* (Sheldrick, 2008 ▶); program(s) used to refine structure: *SHELXL97* (Sheldrick, 2008 ▶); molecular graphics: *ORTEP-3* (Farrugia, 1997 ▶); software used to prepare material for publication: *publCIF* (Westrip, 2010 ▶).

## Supplementary Material

Crystal structure: contains datablocks global, I. DOI: 10.1107/S1600536810048282/cv2800sup1.cif

Structure factors: contains datablocks I. DOI: 10.1107/S1600536810048282/cv2800Isup2.hkl

Additional supplementary materials:  crystallographic information; 3D view; checkCIF report

## Figures and Tables

**Table 1:** Hydrogen-bond geometry (Å, °)

*D*—H⋯*A*	*D*—H	H⋯*A*	*D*⋯*A*	*D*—H⋯*A*
O1—H1*A*⋯N1	0.82	1.85	2.574 (3)	146
C11—H11⋯O1^i^	0.93	2.54	3.464 (4)	175

Symmetry code: (i) 
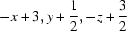
.

**Fig. 1.**
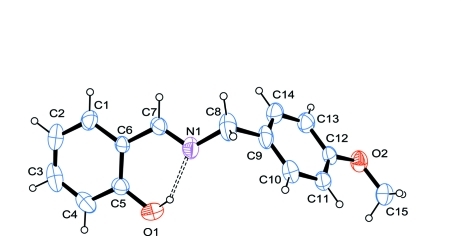
The molecular structure of (I) with displacement ellipsoids for the non-hydrogen atoms drawn at the 30% probability level. Hydrogen bond is shown as a dashed line.

## supplementary crystallographic information

### Comment

Schiff base complexes have gained importance from physiological and pharmacological activities point of view (Adsule *et al.*, 2006; Karthikeyan *et al.*, 2006). As a part of our research focused on the area of transition metal complex-based anticancer agents, the title compound (I) has been prepared as a ligand by Schiff base reaction between 4-methoxybenzylamine and salicylaldehyde.

The molecule of (I) (Fig. 1) adopts a *V*-shape structure. The dihedral angle between the methoxybenzene ring and 2-methyliminophenol moiety is 73.5 (1)°. The 2-methyliminophenol (C1 to C7, N1 and O1) moiety is nearly planar (r.m.s. deviation = 0.021 Å). The methoxybenzene and 2-methyliminophenol groups are located on the opposite side of the C=N bond, showing an *E* configuration. The bond lengths and angles in (I) are normal and comparable with those observed in the related compounds (Phurat *et al.*, 2010; Tariq *et al.*, 2010; Khalaji & Simpson, 2009). Intramolecular O—H···N hydrogen bond (Table 1) generates a S(6) ring (Bernstein *et al.*, 1995).

In the crystal structure, weak intermolecular C—H···O interactions (Table 1) link the molecules into chains propagated in direction [010].

### Experimental

The title compound was prepared according to the method reported in the literature (Kannappan *et al.*, 2005; Phurat *et al.*, 2010). 4-Methoxybenzylamine (2.50 ml.2.63 g, 0.02 mol) was added to a stirred ethanol solution of salicylaldehyde (2.50 ml, 2.86 g, 0.02 mol). The reaction mixture was stirred at reflux for 2 h and then the mixture was allowed to stand at room temperature for 1 week to give yellow cystals suitable for X-ray diffraction analysis.

### Refinement

H-atoms were geometrically positioned (C—H 0.93-0.97 Å, O—H 0.82 Å) and refined using a riding model, with *U*_iso_= 1.2-1.5 U_eq_ of the parent atom. The absolute structure could not be determined and therefore 1,086 Friedel opposites were merged.

### Crystal data

**Table:**

C_15_H_15_NO_2_	*F*(000) = 512
*M**_r_* = 241.28	*D*_x_ = 1.228 Mg m^−^^3^
Orthorhombic, *P*2_1_2_1_2_1_	Mo *K*α radiation, λ = 0.71073 Å
*a* = 5.7190 (8) Å	µ = 0.08 mm^−^^1^
*b* = 12.7229 (19) Å	*T* = 293 K
*c* = 17.936 (3) Å	Needle, yellow
*V* = 1305.0 (3) Å^3^	0.3 × 0.18 × 0.04 mm
*Z* = 4	

### Data collection

**Table:**

Bruker SMART APEXII CCD area-detector diffractometer	1177 reflections with *I* > 2σ(*I*)
Radiation source: Mo Kα	*R*_int_ = 0.040
graphite	θ_max_ = 26.5°, θ_min_ = 2.0°
φ and ω scans	*h* = −7→7
5776 measured reflections	*k* = −15→14
1573 independent reflections	*l* = −20→22

### Refinement

**Table:**

Refinement on *F*^2^	0 restraints
Least-squares matrix: full	H-atom parameters constrained
*R*[*F*^2^ > 2σ(*F*^2^)] = 0.051	*w* = 1/[σ^2^(*F*_o_^2^) + (0.1*P*)^2^] where *P* = (*F*_o_^2^ + 2*F*_c_^2^)/3
*wR*(*F*^2^) = 0.162	(Δ/σ)_max_ < 0.001
*S* = 1.13	Δρ_max_ = 0.15 e Å^−^^3^
1573 reflections	Δρ_min_ = −0.16 e Å^−^^3^
164 parameters	

### Special details

**Table:**

Geometry. All e.s.d.'s (except the e.s.d. in the dihedral angle between two l.s. planes) are estimated using the full covariance matrix. The cell e.s.d.'s are taken into account individually in the estimation of e.s.d.'s in distances, angles and torsion angles; correlations between e.s.d.'s in cell parameters are only used when they are defined by crystal symmetry. An approximate (isotropic) treatment of cell e.s.d.'s is used for estimating e.s.d.'s involving l.s. planes.

### Fractional atomic coordinates and isotropic or equivalent isotropic displacement parameters (Å^2^)

**Table:**

	*x*	*y*	*z*	*U*_iso_*/*U*_eq_	
C1	0.7854 (7)	0.1595 (3)	0.54878 (17)	0.0795 (9)	
H1	0.642	0.183	0.5308	0.095*	
C2	0.8661 (10)	0.0614 (3)	0.5283 (2)	0.1029 (14)	
H2	0.7787	0.0191	0.4965	0.123*	
C3	1.0749 (9)	0.0274 (3)	0.5552 (3)	0.1095 (15)	
H3	1.1283	−0.039	0.5417	0.131*	
C4	1.2087 (7)	0.0879 (3)	0.6015 (2)	0.0909 (11)	
H4	1.3499	0.0627	0.6199	0.109*	
C5	1.1300 (5)	0.1875 (2)	0.62042 (17)	0.0668 (8)	
C6	0.9144 (5)	0.2235 (2)	0.59563 (14)	0.0570 (6)	
C7	0.8246 (5)	0.3261 (2)	0.61737 (18)	0.0675 (7)	
H7	0.6806	0.3482	0.599	0.081*	
C8	0.8369 (7)	0.4881 (3)	0.6807 (3)	0.1079 (14)	
H8A	0.7999	0.4889	0.7335	0.129*	
H8B	0.6929	0.4989	0.6532	0.129*	
C9	1.0065 (6)	0.5755 (3)	0.6632 (2)	0.0814 (10)	
C10	1.1673 (7)	0.6086 (3)	0.71468 (18)	0.0783 (9)	
H10	1.1688	0.5775	0.7616	0.094*	
C11	1.3269 (6)	0.6865 (2)	0.69908 (16)	0.0695 (8)	
H11	1.4343	0.7077	0.735	0.083*	
C12	1.3254 (6)	0.7330 (2)	0.62925 (15)	0.0642 (7)	
C13	1.1673 (8)	0.7010 (3)	0.57680 (19)	0.0836 (10)	
H13	1.1667	0.7319	0.5298	0.1*	
C14	1.0094 (7)	0.6231 (3)	0.5935 (2)	0.0892 (11)	
H14	0.9025	0.6018	0.5575	0.107*	
C15	1.6382 (7)	0.8487 (3)	0.6610 (2)	0.0891 (10)	
H15A	1.73	0.904	0.6394	0.134*	
H15B	1.7389	0.7919	0.6754	0.134*	
H15C	1.5573	0.8749	0.704	0.134*	
N1	0.9379 (4)	0.3862 (2)	0.66067 (15)	0.0790 (7)	
O1	1.2662 (4)	0.2479 (2)	0.66466 (15)	0.0979 (8)	
H1A	1.1921	0.2997	0.6782	0.147*	
O2	1.4738 (5)	0.81229 (18)	0.60808 (11)	0.0847 (7)	

### Atomic displacement parameters (Å^2^)

**Table:**

	*U*^11^	*U*^22^	*U*^33^	*U*^12^	*U*^13^	*U*^23^
C1	0.082 (2)	0.070 (2)	0.0865 (18)	−0.0248 (18)	−0.0068 (17)	0.0110 (16)
C2	0.129 (4)	0.069 (2)	0.110 (3)	−0.037 (3)	0.019 (3)	−0.015 (2)
C3	0.138 (4)	0.055 (2)	0.135 (3)	−0.009 (3)	0.063 (3)	−0.006 (2)
C4	0.079 (2)	0.075 (2)	0.119 (3)	0.015 (2)	0.026 (2)	0.031 (2)
C5	0.0600 (15)	0.0640 (17)	0.0764 (16)	−0.0006 (15)	0.0024 (14)	0.0127 (14)
C6	0.0554 (14)	0.0459 (14)	0.0696 (14)	−0.0079 (12)	0.0013 (12)	0.0121 (12)
C7	0.0515 (13)	0.0542 (16)	0.0968 (18)	−0.0058 (14)	0.0057 (14)	0.0154 (15)
C8	0.091 (2)	0.068 (2)	0.165 (3)	−0.010 (2)	0.043 (3)	−0.030 (2)
C9	0.081 (2)	0.0504 (17)	0.113 (2)	0.0010 (16)	0.026 (2)	−0.0255 (18)
C10	0.099 (2)	0.0543 (16)	0.0814 (18)	0.0031 (19)	0.0169 (18)	−0.0043 (15)
C11	0.0839 (19)	0.0552 (16)	0.0694 (16)	0.0009 (16)	−0.0002 (15)	−0.0039 (13)
C12	0.0789 (18)	0.0457 (14)	0.0680 (14)	0.0075 (16)	0.0052 (14)	−0.0091 (12)
C13	0.108 (3)	0.072 (2)	0.0708 (16)	0.004 (2)	−0.0044 (18)	−0.0100 (15)
C14	0.095 (2)	0.073 (2)	0.099 (2)	0.001 (2)	−0.008 (2)	−0.030 (2)
C15	0.097 (2)	0.070 (2)	0.100 (2)	−0.015 (2)	0.008 (2)	−0.0039 (17)
N1	0.0726 (15)	0.0577 (15)	0.1068 (17)	−0.0101 (14)	0.0126 (15)	−0.0071 (14)
O1	0.0699 (13)	0.111 (2)	0.1132 (17)	0.0012 (16)	−0.0258 (14)	0.0017 (16)
O2	0.1129 (18)	0.0647 (13)	0.0765 (12)	−0.0104 (13)	0.0065 (13)	0.0056 (11)

### Geometric parameters (Å, °)

**Table:**

C1—C2	1.381 (6)	C8—H8B	0.97
C1—C6	1.383 (4)	C9—C10	1.369 (5)
C1—H1	0.93	C9—C14	1.390 (5)
C2—C3	1.359 (7)	C10—C11	1.376 (5)
C2—H2	0.93	C10—H10	0.93
C3—C4	1.366 (6)	C11—C12	1.385 (4)
C3—H3	0.93	C11—H11	0.93
C4—C5	1.387 (5)	C12—C13	1.367 (5)
C4—H4	0.93	C12—O2	1.372 (4)
C5—O1	1.352 (4)	C13—C14	1.374 (5)
C5—C6	1.389 (4)	C13—H13	0.93
C6—C7	1.456 (4)	C14—H14	0.93
C7—N1	1.268 (4)	C15—O2	1.413 (4)
C7—H7	0.93	C15—H15A	0.96
C8—N1	1.464 (4)	C15—H15B	0.96
C8—C9	1.509 (5)	C15—H15C	0.96
C8—H8A	0.97	O1—H1A	0.82
			
C2—C1—C6	121.0 (4)	C10—C9—C14	117.7 (3)
C2—C1—H1	119.5	C10—C9—C8	121.2 (4)
C6—C1—H1	119.5	C14—C9—C8	121.0 (4)
C3—C2—C1	119.1 (4)	C9—C10—C11	122.0 (3)
C3—C2—H2	120.4	C9—C10—H10	119
C1—C2—H2	120.4	C11—C10—H10	119
C2—C3—C4	121.9 (4)	C10—C11—C12	119.2 (3)
C2—C3—H3	119	C10—C11—H11	120.4
C4—C3—H3	119	C12—C11—H11	120.4
C3—C4—C5	118.8 (4)	C13—C12—O2	116.0 (3)
C3—C4—H4	120.6	C13—C12—C11	119.9 (3)
C5—C4—H4	120.6	O2—C12—C11	124.1 (3)
O1—C5—C4	118.5 (3)	C12—C13—C14	120.0 (3)
O1—C5—C6	120.8 (3)	C12—C13—H13	120
C4—C5—C6	120.7 (3)	C14—C13—H13	120
C1—C6—C5	118.3 (3)	C13—C14—C9	121.2 (3)
C1—C6—C7	120.1 (3)	C13—C14—H14	119.4
C5—C6—C7	121.6 (3)	C9—C14—H14	119.4
N1—C7—C6	121.6 (3)	O2—C15—H15A	109.5
N1—C7—H7	119.2	O2—C15—H15B	109.5
C6—C7—H7	119.2	H15A—C15—H15B	109.5
N1—C8—C9	110.4 (3)	O2—C15—H15C	109.5
N1—C8—H8A	109.6	H15A—C15—H15C	109.5
C9—C8—H8A	109.6	H15B—C15—H15C	109.5
N1—C8—H8B	109.6	C7—N1—C8	118.9 (3)
C9—C8—H8B	109.6	C5—O1—H1A	109.5
H8A—C8—H8B	108.1	C12—O2—C15	117.8 (2)
			
C6—C1—C2—C3	0.5 (5)	C14—C9—C10—C11	0.4 (5)
C1—C2—C3—C4	−0.6 (6)	C8—C9—C10—C11	178.5 (3)
C2—C3—C4—C5	−1.1 (6)	C9—C10—C11—C12	−0.1 (5)
C3—C4—C5—O1	−178.3 (3)	C10—C11—C12—C13	−0.3 (4)
C3—C4—C5—C6	2.9 (5)	C10—C11—C12—O2	179.0 (3)
C2—C1—C6—C5	1.3 (4)	O2—C12—C13—C14	−179.0 (3)
C2—C1—C6—C7	−179.2 (3)	C11—C12—C13—C14	0.3 (5)
O1—C5—C6—C1	178.2 (3)	C12—C13—C14—C9	0.0 (5)
C4—C5—C6—C1	−3.0 (4)	C10—C9—C14—C13	−0.3 (5)
O1—C5—C6—C7	−1.3 (4)	C8—C9—C14—C13	−178.4 (3)
C4—C5—C6—C7	177.5 (3)	C6—C7—N1—C8	−179.6 (3)
C1—C6—C7—N1	179.4 (3)	C9—C8—N1—C7	−125.6 (4)
C5—C6—C7—N1	−1.0 (4)	C13—C12—O2—C15	179.2 (3)
N1—C8—C9—C10	−89.5 (4)	C11—C12—O2—C15	0.0 (4)
N1—C8—C9—C14	88.6 (5)		

### Hydrogen-bond geometry (Å, °)

**Table:**

*D*—H···*A*	*D*—H	H···*A*	*D*···*A*	*D*—H···*A*
O1—H1A···N1	0.82	1.85	2.574 (3)	146
C11—H11···O1^i^	0.93	2.54	3.464 (4)	175

Symmetry codes: (i) −*x*+3, *y*+1/2, −*z*+3/2.

## References

[bb1] Adsule, S., Barve, V., Chen, D., Ahmed, F., Dou, Q. P., Padhye, S. & Sarkar, F. H. (2006). *J. Med. Chem.* **49**, 7242–7246. 10.1021/jm060712l 17125278

[bb2] Bernstein, J., Davis, R. E., Shimoni, L. & Chang, N.-L. (1995). *Angew. Chem. Int. Ed. Engl.* **34**, 1555–1573.

[bb3] Bruker (2008). *APEX2*, *SAINT* and *SADABS* Bruker AXS Inc., Madison, Wisconsin, USA.

[bb4] Farrugia, L. J. (1997). *J. Appl. Cryst.* **30**, 565.

[bb5] Kannappan, R., Tanase, S., Mutikainen, I., Turpeinen, U. & Reedijk, J. (2005). *Inorg. Chim. Acta*, **358**, 383–388.

[bb6] Karthikeyan, M. S., Prasad, D. J., Poojary, B., Bhat, K. S., Holla, B. S. & Kumari, N. S. (2006). *Bioorg. Med. Chem.* **14**, 7482–7489. 10.1016/j.bmc.2006.07.015 16879972

[bb7] Khalaji, A. D. & Simpson, J. (2009). *Acta Cryst.* E**65**, o362. 10.1107/S1600536809001871 PMC2968393 21581960

[bb8] Phurat, C., Teerawatananond, T. & Muangsin, N. (2010). *Acta Cryst.* E**66**, o2310. 10.1107/S1600536810031508 PMC3007859 21588659

[bb9] Sheldrick, G. M. (2008). *Acta Cryst.* A**64**, 112–122. 10.1107/S0108767307043930 18156677

[bb10] Tariq, M. I., Ahmad, S., Tahir, M. N., Sarfaraz, M. & Hussain, I. (2010). *Acta Cryst.* E**66**, o1561. 10.1107/S160053681001932X PMC3006706 21587804

[bb11] Westrip, S. P. (2010). *J. Appl. Cryst.* **43**, 920–925.

